# Topoisomerase II Inhibitors Induce DNA Damage-Dependent Interferon Responses Circumventing Ebola Virus Immune Evasion

**DOI:** 10.1128/mBio.00368-17

**Published:** 2017-04-04

**Authors:** Priya Luthra, Sebastian Aguirre, Benjamin C. Yen, Colette A. Pietzsch, Maria T. Sanchez-Aparicio, Bersabeh Tigabu, Lorraine K. Morlock, Adolfo García-Sastre, Daisy W. Leung, Noelle S. Williams, Ana Fernandez-Sesma, Alexander Bukreyev, Christopher F. Basler

**Affiliations:** a Center for Microbial Pathogenesis, Institute for Biomedical Sciences, Georgia State University, Atlanta, Georgia, USA; b Department of Microbiology, Icahn School of Medicine at Mount Sinai, New York, New York, USA; c Department of Pathology, Galveston National Laboratory, The University of Texas Medical Branch at Galveston, Galveston, Texas, USA; d Department of Biochemistry, UT Southwestern Medical Center, Dallas, Texas, USA; e Global Health and Emergent Pathogens Institute, Icahn School of Medicine at Mount Sinai, New York, New York, USA; f Department of Medicine, Division of Infectious Diseases, Icahn School of Medicine at Mount Sinai, New York, New York, USA; g Department of Pathology and Immunology, Washington University School of Medicine, St. Louis, Missouri, USA; h Department of Microbiology & Immunology, University of Texas Medical Branch at Galveston, Galveston, Texas, USA; i Galveston National Laboratory, University of Texas Medical Branch at Galveston, Galveston, Texas, USA; University of Pittsburgh School of Medicine

**Keywords:** ATM signaling, DNA damage, innate immune responses, cGAS-STING pathway, Ebola virus

## Abstract

Ebola virus (EBOV) protein VP35 inhibits production of interferon alpha/beta (IFN) by blocking RIG-I-like receptor signaling pathways, thereby promoting virus replication and pathogenesis. A high-throughput screening assay, developed to identify compounds that either inhibit or bypass VP35 IFN-antagonist function, identified five DNA intercalators as reproducible hits from a library of bioactive compounds. Four, including doxorubicin and daunorubicin, are anthracycline antibiotics that inhibit topoisomerase II and are used clinically as chemotherapeutic drugs. These compounds were demonstrated to induce IFN responses in an ATM kinase-dependent manner and to also trigger the DNA-sensing cGAS-STING pathway of IFN induction. These compounds also suppress EBOV replication *in vitro* and induce IFN in the presence of IFN-antagonist proteins from multiple negative-sense RNA viruses. These findings provide new insights into signaling pathways activated by important chemotherapy drugs and identify a novel therapeutic approach for IFN induction that may be exploited to inhibit RNA virus replication.

## IMPORTANCE

Ebola virus and other emerging RNA viruses are significant but unpredictable public health threats. Therapeutic approaches with broad-spectrum activity could provide an attractive response to such infections. We describe a novel assay that can identify small molecules that overcome Ebola virus-encoded innate immune evasion mechanisms. This assay identified as hits cancer chemotherapeutic drugs, including doxorubicin. Follow-up studies provide new insight into how doxorubicin induces interferon (IFN) responses, revealing activation of both the DNA damage response kinase ATM and the DNA sensor cGAS and its partner signaling protein STING. The studies further demonstrate that the ATM and cGAS-STING pathways of IFN induction are a point of vulnerability not only for Ebola virus but for other RNA viruses as well, because viral innate immune antagonists consistently fail to block these signals. These studies thereby define a novel avenue for therapeutic intervention against emerging RNA viruses.

## INTRODUCTION

Emerging RNA viruses are an ongoing but unpredictable threat to public health. A potential answer to this threat would be a broad-spectrum therapeutic approach targeting host pathways in a manner that would inhibit the diverse virus families that are likely to cause outbreaks in the human population. Ebola virus (EBOV), a member of the family *Filoviridae*, represents one such threat. EBOV has repeatedly been associated with outbreaks of highly lethal human disease (1). The need for antifiloviral therapies is highlighted by the EBOV outbreak in West Africa that caused more than 28,000 infections and 11,000 deaths (2). One potential avenue for therapeutic intervention would be to target the innate immune evasion functions of EBOV and other emerging RNA viruses. Filoviruses inhibit type I interferon (IFN) responses at multiple levels (3–13). One major mechanism of EBOV IFN suppression is carried out by the VP35 protein, which inhibits signaling by the RIG-I-like receptors (RLR), RIG-I and MDA5 (11, 14, 15). Suppression of RLR signaling not only prevents IFN production but can also block the induction of a subset of antiviral genes that can be expressed as a direct result of RLR signaling and independently of IFN production. Further, for EBOV, VP35 inhibition of RLR signaling suppresses dendritic cell (DC) maturation, likely contributing to suppression of adaptive immune responses (15, 16). That the IFN-antagonist function of VP35 represents a potential therapeutic approach for Ebola virus disease (EVD) is suggested by the fact that recombinant EBOVs engineered to lack VP35 IFN-antagonist function exhibit severe attenuation in cell culture and *in vivo* (12, 17, 18).

Numerous RNA viruses target RLR pathways and other aspects of the IFN response (as reviewed in references 19 and 20). Identification of an IFN-inducing pathway that bypasses blocks imposed by EBOV and other RNA viruses could serve as a means to generally suppress RNA virus replication. Candidate pathways include cellular DNA-sensing pathways that lead to IFN gene expression; viruses that lack a DNA genome and do not produce DNA products of replication may not have evolved mechanisms to suppress the DNA-induced responses. Among DNA-sensing mechanisms, the kinase ATM, which is activated in response to DNA breaks, has been identified as promoting IFN production, although relevant downstream signaling events that lead to IFN production remain incompletely defined (21–26). Another particularly well-characterized DNA sensing pathway is the cGAS-STING pathway, in which cytoplasmic DNA binds and activates the enzyme cGAS, triggering its generation of the cyclic dinucleotide (CDN) cyclic GMP-AMP (cGAMP) (27, 28). CDN activates signaling through STING to trigger IFN production (29–31). The cGAS-STING pathway has also been implicated in triggering IFN production in response to DNA damage (22).

Anthracycline antibiotics are a class of compounds which includes commonly used cancer chemotherapy drugs such as doxorubicin, which, although highly effective in killing tumor cells, is limited in its usage due to its cardiotoxicity (32). These compounds intercalate DNA, inhibit type II topoisomerase, and trigger the DNA damage response (33, 34). One interesting but relatively understudied effect of these compounds on cells is induction of IFN responses; induction of such responses has been proposed to modulate immune responses that may influence the antitumor effects of doxorubicin (35, 36).

Here, we developed and optimized a high-throughput screening (HTS) assay in a 384-well format with the initial goal of identifying compounds that induce IFN in the presence of EBOV VP35 protein. A screen of 2,080 bioactive compounds identified DNA-intercalating chemotherapeutic agents such as doxorubicin and daunorubicin as reproducible activators of the IFN-β promoter in the presence of VP35. These drugs are DNA topoisomerase II poisons that intercalate DNA (37). We demonstrate that these drugs can activate the IFN-β promoter via either the DNA damage response-associated kinase ATM or the cGAS-STING pathway, that activation of the ATM pathway requires the presence of DNA topoisomerase II, and that VP35 blocks neither pathway. The compounds are further demonstrated to suppress EBOV replication and to activate an IFN response in the presence of IFN antagonists from several different RNA viruses. These observations identify new host pathways that are activated by anthracycline chemotherapeutic drugs, define mechanisms by which these pathways are activated, and suggest that the DNA damage response and DNA-sensing pathways could be exploited to treat infections by EBOV and other RNA viruses.

## RESULTS

### An HTS assay to identify small-molecule inhibitors of VP35.

A 293T-based stable cell line with a firefly luciferase reporter gene under the control of the IFN-β promoter (293T-FF) was transduced with a lentivirus that expresses from a single mRNA both VP35 and green fluorescent protein (GFP) (15). This yielded the cell line VP35-FF. In this cell line, an internal ribosomal entry site separates the open reading frames for VP35 and GFP such that the two proteins are translated as distinct polypeptides. Alternatively, the reporter cell line was transduced with an “empty-vector” lentivirus that expresses GFP alone (control-FF). Clonal VP35-FF and control-FF cell lines were obtained by sorting for GFP expression (see Fig. S1A in the supplemental material). Upon infection with Sendai virus (SeV), a known activator of RLR signaling and of the IFN-β promoter, a strong upregulation of luciferase expression was detected in the control-FF cells, whereas the VP35-FF cells exhibited little response to infection, reflecting VP35 inhibition of RLR signaling and IFN-β promoter activation (Fig. S1B). Examination of endogenous mRNA levels for IFN-β and interferon stimulated gene 54 (ISG54) yielded parallel results (Fig. S1C and D), demonstrating that the reporter gene accurately reflects the status of the endogenous IFN response.

**FIG 1:**
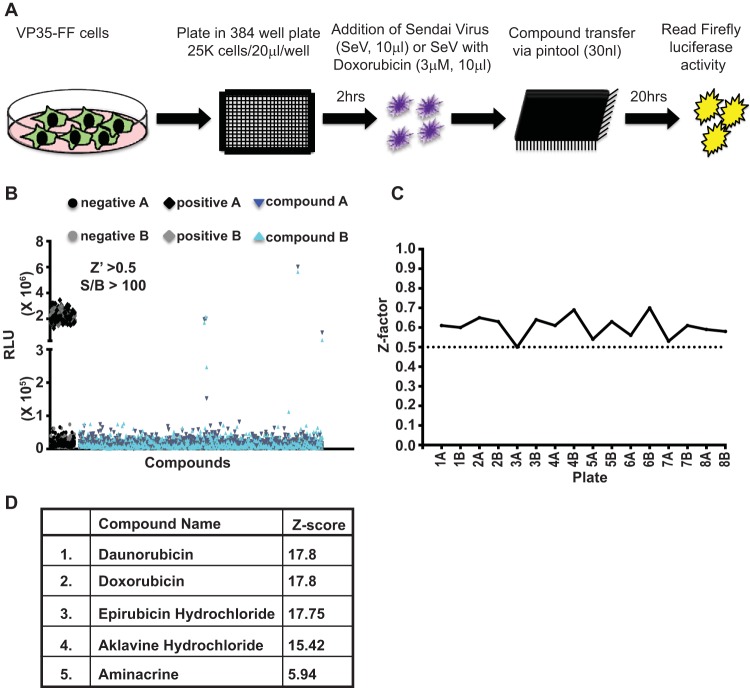
Establishing a high-throughput screening (HTS) assay to identify inhibitors of VP35. (A) Schematic for high-throughput screening assay of VP35 function. Stable VP35 cells were dispensed in 384-well plates using an automated dispenser. Two hours later, cells were treated with SeV (negative control) or SeV plus doxorubicin (positive control). Compound addition was done via pin tool transfer. Twenty hours posttreatment, a luciferase assay was performed. (B) Results of HTS. A total of 2,080 bioactive compounds were screened (8 screening plates). Each screening plate was run in duplicate (indicated by A or B). Data points indicate relative luciferase units (RLU) for each sample. Controls were as described for panel A. The overall Z factor for the screen was greater than 0.5, and the signal-to-background ratio (S/B) was greater than 100. (C) Z values for each 384-well plate in the pilot screen are plotted. (D) The 5 hits identified by the pilot screen that had a Z score greater than 5 in both replicates are listed along with the average Z score for the two replicates. See also Fig. S1 in the supplemental material.

10.1128/mBio.00368-17.2 FIG S1  Related to Fig. 1. Establishment of a high-throughput screening assay to identify inhibitors of VP35. (A) Generation of stable VP35 cells. HEK293T cells stably transfected with a firefly luciferase gene under the control of the IFN-β promoter (293T-FF) were transduced with lentiviruses that express either GFP alone (control-FF) or GFP and VP35 (VP35-FF) to generate stable cell lines for HTS assays. The Western blot shows the expression of GFP and VP35 in the stable cell lines. (B) Characterization of stable cell lines. Control-FF or VP35-FF cells were plated on 384-well plates and, the next day, mock treated or treated with SeV or doxorubicin (1 µM). Eighteen hours posttreatment, luciferase activity was determined. Control-FF or VP35-FF cells were treated with doxorubicin (1 µM) or infected with Sendai virus. Twelve hours posttreatment, total RNA was extracted using Trizol. qRT-PCR was performed for endogenous IFN-β (C) or ISG54 (D) mRNA levels, which were normalized to β-actin mRNA. (E) The HTS assay in a 384-well format. The VP35 cells were plated in 384-well plates. Two hours later, cells were infected with SeV in the presence of either DMSO (SeV + DMSO) or 3 µM doxorubicin (SeV + Doxo). Twenty hours later, luciferase activity was measured. (F) Knockdown of VP35 restores responsiveness of cells to SeV infection. The VP35 cells were mock transfected (untreated) or transfected with scrambled or VP35-specific (si349 and si219) small interfering RNAs. Seventy-two hours posttransfection, cells were mock treated, treated with doxorubicin (doxo), or infected with SeV. Twenty hours later, luciferase activity was measured. The Western blot demonstrates knockdown of VP35 expression. Download FIG S1, EPS file, 3.3 MB. Copyright © 2017 Luthra et al. 2017 Luthra et al. This content is distributed under the terms of the Creative Commons Attribution 4.0 International license.

For an HTS screen, it was desirable to identify a positive-control compound that would induce an IFN response in the presence of VP35. However, no small-molecule inhibitor of VP35 IFN-antagonist function has been described. We assessed the FDA-approved chemotherapeutic drug doxorubicin, which has been reported to induce an IFN response and to stimulate IFN regulatory factor 3 (IRF-3) phosphorylation by an incompletely defined mechanism (38, 39). Doxorubicin activated the IFN-β promoter in the presence or absence of VP35 (Fig. S1B). Doxorubicin also stimulated an endogenous IFN response in either reporter cell line as indicated by upregulation of IFN-β and ISG54 mRNAs (Fig. S1C and D). As doxorubicin induced IFN in the presence or absence of VP35 and IFN induction occurred in the absence of an RLR activator such as SeV, IFN induction by doxorubicin is likely through a signaling pathway that is not blocked by VP35.

An HTS assay based on the VP35-FF cell line was developed to allow identification of additional small molecules that induce an IFN response in the presence of VP35 (Fig. 1A). Briefly, VP35-FF cells were plated in 384-well plates, allowed to rest for 2 h, and infected with SeV in the presence of either diluent (0.1% dimethyl sulfoxide [DMSO]) or 3 µM doxorubicin. Immediately afterward, compounds were added via pin tool transfer. Twenty hours postaddition, luciferase activity was measured. A representative pilot study compared VP35-FF cells that were infected with SeV and treated with DMSO (SeV + DMSO) to the same cells infected with SeV and treated with 3 µM doxorubicin (SeV + doxorubicin). Comparison of the two conditions yielded an 83-fold induction by doxorubicin over the DMSO control and a Z factor of 0.7 (Fig. S1E). A Z factor value of >0.5 indicates a high-quality screening assay (40). To establish that inhibition of VP35 can result in activation of IFN-β promoter by SeV in the VP35-FF cells, we utilized previously described VP35 small interfering RNAs (siRNAs) si349 and si219 (41). VP35-FF cells that were transfected with the siRNAs to VP35 mRNA had reduced VP35 expression levels compared to a scrambled siRNA (Fig. S1F). Following SeV infection, little IFN-β promoter activation was observed in the scrambled siRNA-treated cells, but IFN-β responses were stimulated by SeV infection upon VP35 knockdown (Fig. S1F). Doxorubicin-mediated activation of IFN-β promoter was not impaired by VP35 knockdown.

Using the optimized conditions for the 384-well format, we screened a library of 2,080 bioactive compounds (Fig. 1B). The eight library plates were each screened in duplicate, and the Z factor for each plate was ≥0.5 (Fig. 1C). The Z scores, which indicate how many standard deviations a given value is from the mean, were calculated for each compound, and those compounds with Z scores of ≥5 in both replicates were classified as hits. This resulted in 5 hits. Perhaps unsurprisingly, the hits included doxorubicin. Strikingly, three other hits were daunorubicin, epirubicin, and aklavine hydrochloride, all anthracycline antibiotics that are structurally very similar to doxorubicin. Doxorubicin, daunorubicin, and epirubicin are used as chemotherapeutic drugs for cancer (42, 43). The last hit was aminacrine (9-aminoacridine), a fluorescent dye used clinically as a topical antiseptic and experimentally as a mutagen due to its interaction with DNA (44) (Fig. 1D).

### Doxorubicin and daunorubicin stimulate production of IFN-α/β.

**FIG 2:**
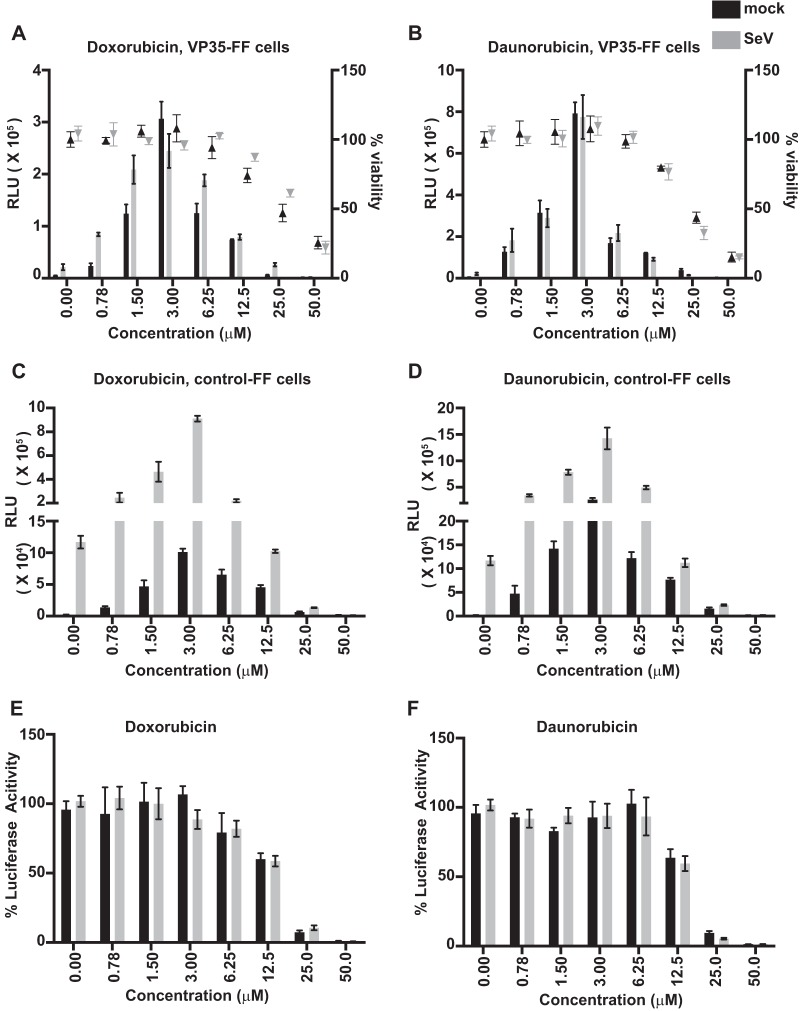
IFN induction and cytotoxicity of doxorubicin and daunorubicin in reporter cell lines. **(**A to D) (A and B) Dose response in VP35 cells for activation of the IFN-β reporter luciferase reporter gene (bars) and for cytotoxicity (triangles) of doxorubicin (A) or daunorubicin (B). (C and D) Dose response in control-FF cells for activation of the IFN-β reporter luciferase reporter gene by doxorubicin (C) and daunorubicin (D). (E and F) The effect of doxorubicin (E) and daunorubicin (F) on expression of a constitutively expressed firefly luciferase gene. Percent luciferase activity is relative to that with no drug treatment. Data represent means ± standard deviations and are representative of three independent experiments. RLU, relative luciferase units.

**FIG 3:**
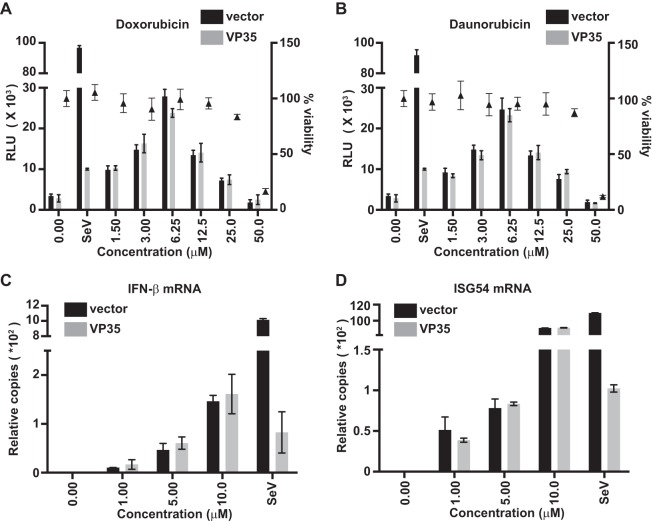
Induction of an IFN response by doxorubicin and daunorubicin is not cell type specific. Dose response for activation of the IFN-β reporter gene (bars) and for cytotoxicity (triangles) of doxorubicin (A) or daunorubicin (B) in A549 cells transfected with empty vector (vector) or VP35. Reverse transcription-quantitative polymerase chain reaction (qRT-PCR) was performed for endogenous IFN-β (C) or ISG54 (D) mRNA levels in A549 cells transfected with empty vector (vector) or VP35 and treated with doxorubicin. The RNA was isolated 12 h after treatment with the indicated concentrations of drug, and levels were normalized to levels of β-actin mRNA. Data represent means ± standard deviations and are representative of three independent experiments.

We asked how anthracyclines stimulate an IFN response and why this stimulation is not blocked by VP35, choosing doxorubicin and daunorubicin for this analysis. First, the doses required for IFN-β promoter activation and for cytotoxicity were determined in both VP35-FF (Fig. 2A and B) and control-FF (Fig. 2C and D) cells in the absence or presence of SeV infection. Both compounds were toxic to cells at higher concentrations (25 to 50 μM), consistent with their use as cancer drugs. However, each activated the IFN-β promoter at concentrations far below cytotoxic levels, with as little as 780 nM inducing luciferase expression and with peak stimulation at 3 µM in either the VP35-FF cells (Fig. 2A and B) or the control-FF cells (Fig. 2C and D). To confirm that neither doxorubicin nor daunorubicin nonspecifically enhances luciferase activity, cells transfected with a reporter plasmid from which firefly luciferase is constitutively expressed were treated with different doses of each drug. No significant stimulation was observed, demonstrating specificity toward the IFN-β promoter reporter gene (Fig. 2E and F). To determine whether the IFN-β induction occurs in other cell types, we transiently transfected A549 cells with an IFN-β–luciferase reporter gene and with empty vector or VP35 expression plasmid and treated them with different doses of doxorubicin or daunorubicin. Each drug induced reporter gene expression at noncytotoxic doses in the absence or presence of VP35 (Fig. 3A and B). Each drug also induced expression of the endogenous IFN-β and ISG54 mRNAs regardless of whether VP35 was expressed (Fig. 3C and D).

### Doxorubicin and daunorubicin induce IFN by an ATM-dependent mechanism.

**FIG 4:**
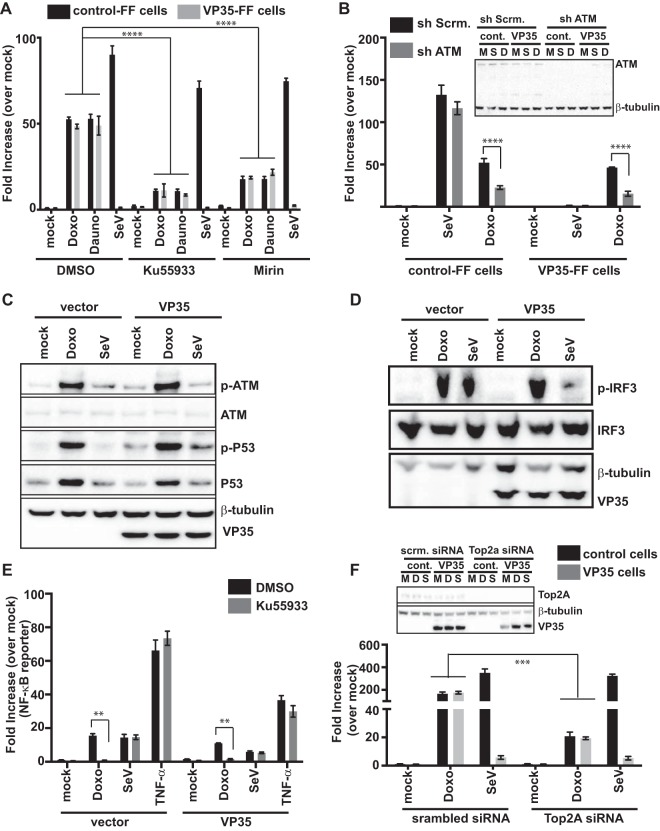
An ATM-dependent IFN response that is not blocked by VP35 is stimulated by doxorubicin and daunorubicin. (A) An IFN-β promoter assay was performed as described above except that cells (control or VP35) were treated with DMSO, ATM kinase inhibitor Ku55933 (10 μM), or mirin (10 μM) for 2 h before doxorubicin (1 μM) or daunorubicin (1 μM) treatment or SeV infection. ****, *P* value < 0.0001 (one-way analysis of variance followed by Tukey’s test). (B) IFN-β promoter reporter gene assays were performed as described above except that cells were transfected with scrambled shRNA (sh Scrm.) or ATM-specific shRNA (sh ATM) plasmids. ****, *P* value < 0.0001 (one-way analysis of variance followed by Tukey’s test). A Western blot for ATM and VP35 is shown in the inset. M, mock treated (medium + DMSO); S, SeV infected; D, doxorubicin (1 μM) treated. (C) Phospho-ATM (S1981), phospho-p53 (S15), total ATM, total p53, and VP35 levels were assessed by Western blotting in HEK293T cells transfected with empty vector or VP35 and mock treated (mock), treated with doxorubicin (Doxo), or infected with SeV (SeV) at 4 h posttreatment. β-Tubulin served as a loading control. (D) Phospho-IRF-3 (p-IRF-3) and total IRF-3 (IRF-3) levels were assessed in HEK293T cells transfected with FLAG-IRF-3 plasmid and either empty vector (vector) or VP35 plasmid. The cells were either mock treated or treated with doxorubicin or infected with SeV for 8 h. β-Tubulin served as a loading control. Total IRF-3 levels were assessed by using anti-FLAG, p-IRF-3 levels were assessed by using anti-p-IRF-3 (Ser396), and VP35 levels were assessed by using anti-VP35 antibodies. (E) NF-κB firefly luciferase reporter gene activity in mock- or VP35-transfected cells that were mock treated (medium + DMSO), treated with doxorubicin, or infected with SeV in the presence or absence of ATM kinase inhibitor Ku55933. Cells treated with 50 ng/ml of TNF-α for 2 h served as a known NF-κB activation control. Fold induction is relative to the mock-treated, vector control. Data represent means ± standard deviations and are representative of three independent experiments. **, *P* value < 0.01. (F) IFN-β reporter gene assays were performed as described above in control cells or VP35 cells but in the presence of scrambled siRNA (scrm.) or Top2A-specific siRNA (Top2a). ***, *P* value < 0.001 (one-way analysis of variance followed by Tukey’s test). The inset shows Western blotting assays to detect Top2A and β-tubulin. M, mock treated; D, doxorubicin treated; S, SeV infected.

Doxorubicin has pleiotropic effects on cells. Among its activities, it is a topoisomerase II poison that intercalates into DNA, resulting in double-strand DNA breaks (DSB) (24, 45). Ataxia-telangiectasia mutated (ATM), a member of the phosphoinositide 3-kinase-like family of serine/threonine protein kinases, is activated in response to DNA DSB (25, 46–48). ATM has also been linked to stimulation of innate immune signaling pathways (26, 49–52). This prompted us to examine the role of ATM in doxorubicin- and daunorubicin-mediated activation of the IFN-β promoter. We treated control-FF or VP35–FF cells with an ATM kinase inhibitor (Ku55933) (53) or with mirin, an inhibitor of the Mre11-Rad50-Nbs1 (MRN)-ATM pathway, which is essential for sensing and signaling in response to double-strand DNA breaks. Mirin prevents MRN-dependent activation of ATM without affecting ATM protein kinase activity and inhibits Mre11-associated exonuclease activity (54). Each inhibitor significantly dampened, in the presence or absence of VP35, the IFN-β promoter activity induced by doxorubicin or daunorubicin compared to DMSO treatment (Fig. 4A). To further implicate the ATM pathway in the response to doxorubicin, short hairpin RNA (shRNA) knockdown of ATM was performed. Relative to a scrambled shRNA, targeting ATM decreased the doxorubicin-mediated IFN induction in control-FF and VP35-FF cells relative to mock-treated controls (Fig. 4B). In contrast, neither pharmacological inhibition nor shRNA knockdown significantly affected SeV-mediated induction of IFN-β promoter activity in control-FF cells (Fig. 4A and B).

Activation of ATM results in its phosphorylation and the phosphorylation of downstream targets, including p53. To further establish that the ATM pathway is active upon doxorubicin treatment, we examined the phosphorylation status of ATM and p53. The phosphorylation of ATM serine 1981 and of p53 serine 15 was assessed after 6 h of treatment with doxorubicin. SeV infection was included as a control. The drug but not SeV resulted in phosphorylation of ATM and p53 in the presence or absence of VP35 (Fig. 4C). Both IRF-3 and NF-κB are transcription factors that contribute to induction of the IFN-β promoter. Doxorubicin treatment also resulted in Ser396 phosphorylation of IRF-3, consistent with its activation. In contrast to the inhibition seen with SeV infection, VP35 did not prevent doxorubicin-induced IRF-3 phosphorylation (Fig. 4D). Further, doxorubicin activated NF-κB-directed gene expression, as assessed by reporter gene assay, and this was impaired by the ATM kinase inhibitor but not by VP35. This was in contrast to the case when SeV or tumor necrosis factor alpha (TNF-α) was used as an NF-κB activator, where ATM inhibition did not affect induction (Fig. 4E). Cumulatively, these data suggest that doxorubicin and related compounds induce IFN responses, at least in part, via an ATM-dependent pathway and that VP35 expression does not block this pathway.

Doxorubicin targets topoisomerase IIα (Top2A) and generates stabilized DNA-topoisomerase II covalent complexes (33). Decreasing levels of Top2A render cells resistant to killing by doxorubicin (34, 55). To examine whether decreased Top2A levels influence activation of the IFN-β promoter, small interfering RNA was used to decrease Top2A expression. Downregulation of Top2A expression reduced IFN-β promoter activation by doxorubicin in both the presence and the absence of VP35 but did not have any effect on SeV-mediated activation of IFN (Fig. 4F). Cumulatively, these data are consistent with a model whereby doxorubicin inhibition of Top2A activates ATM, which leads to IFN induction.

### The cGAS-STING axis can also contribute to doxorubicin-mediated activation of interferon responses.

DNA damage or infection may lead to generation of cytosolic DNA (cDNA) that can trigger IFN induction (56, 57). The endoplasmic-reticulum-resident protein stimulator of interferon genes (STING) is required for the initiation of signaling leading to IFN production upon detection of cytosolic DNA and also serves as a direct receptor for the detection of DNA (29, 58). cGAS (cyclic GMP-AMP synthase) is an enzyme that recognizes DNA in the cytoplasm and generates a unique cGAMP isomer, with one 2′-5′ phosphodiester bond and one 3′-5′ phosphodiester bond, that binds and activates STING (59). Interestingly, the cGAS-STING pathway has been implicated in induction of IFN by cellular DNA damage (22). cGAS and STING were not detectable by Western blotting in the 293T cell-based control-FF or VP35-FF cell lines (Fig. S2A), which is consistent with a previous report that 293T cells lack cGAS and STING (60). Therefore, to address the potential role of these proteins to signal in response to doxorubicin, 293T-based reporter cell lines were stably transduced with lentiviruses that express wild-type human STING (STING-FF cells) (29, 61–63).

**FIG 5:**
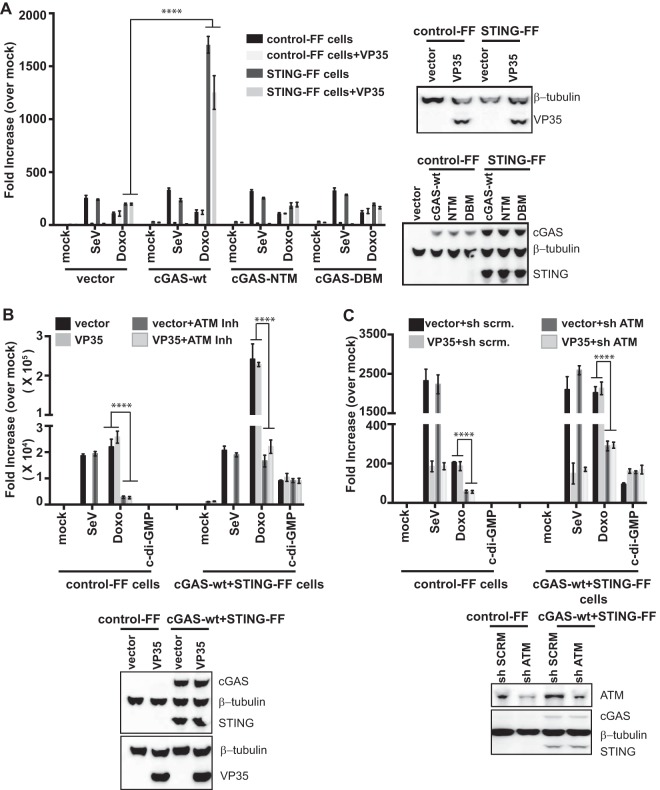
cGAS and STING enhance IFN induction by doxorubicin. (A) IFN-β reporter gene assays were performed as described above in control cells or cell lines with stable expression of STING. These were transfected with empty vector, cGAS-wt, NTase mutant cGAS (cGAS-NTM), or DNA binding cGAS mutant (cGAS-DBM). Some cells were also transfected with VP35 plasmid, as indicated. The next day, cells were mock treated, treated with doxorubicin (Doxo), or infected with SeV. Twenty hours later, reporter gene activity was measured. The Western blot indicates expression of STING, cGAS, VP35, and β-tubulin as a loading control. (B and C) IFN-β reporter control cells or cells stably expressing STING and wt-cGAS were transduced with empty vector or VP35-expressing lentiviruses. Three days later, cells were pretreated with ATM kinase inhibitor Ku55933 (10 μM) for 2 h (B) or transfected with scrambled short hairpin RNA (sh scrnm.) or ATM-specific short hairpin RNA plasmid (sh ATM) to knock down ATM expression (C) and mock treated (medium + DMSO), treated with doxorubicin (Doxo, 3 μM), induced with c-di-GMP (20 μg), or infected with SeV. Twenty hours later, IFN-β reporter activation was measured by luciferase assay. The Western blots show expression of STING, cGAS, ATM, VP35, and β-tubulin. ****, *P* value < 0.0001 (one-way analysis of variance followed by Tukey’s test). Error bars represent means ± standard deviations, and values are representative of three independent experiments. See also Fig. S2 and S3 in the supplemental material.

10.1128/mBio.00368-17.3 FIG S2  Related to Fig. 5. STING enhances IFN induction by doxorubicin. (A) Western blot for endogenous STING and cGAS in control-FF, VP35-FF, A549, and dendritic (DC) cells. (B) Steady-state levels of IFN-β in human wild-type fibroblasts (healthy control) or ATM-deficient fibroblasts (AT cells). Human wild-type fibroblasts (healthy control) (C) or ATM-deficient fibroblasts (AT cells) (D) were transduced with vector control or VP35-expressing lentiviruses. The cells were treated with doxorubicin, c-di-GMP, or SeV. The RNA was harvested at the indicated times, and endogenous levels of IFN-β were determined. Primary human monocyte-derived DCs were transduced with lentiviruses expressing vector control or VP35 Ebola virus protein (control or VP35). Seventy-two hours posttransduction, the cells were treated with doxorubicin (1 μM) or c-di-GMP or infected with SeV. After the indicated times, qRT-PCR was performed for endogenous IFN-β (E) or ISG54 (F) mRNA levels and values were normalized to β-actin mRNA levels. Download FIG S2, EPS file, 2.5 MB. Copyright © 2017 Luthra et al. 2017 Luthra et al. This content is distributed under the terms of the Creative Commons Attribution 4.0 International license.

To validate the STING cell lines, empty vector or VP35 was transfected along with expression plasmids for wild-type cGAS (cGAS-wt), a cGAS nucleotidyltransferase G212A/S213A mutant (cGAS-NTase), or a cGAS DNA binding mutant C396A/C397A (cGAS-DBM). cGAS-NTM has mutations in the active site of cGAS and abolishes production of cGAMPs by cGAS (64, 65). cGAS-DBM was generated by mutating two cysteine residues of the zinc-binding site so as to abolish DNA-induced NTase activity (65, 66). The following day, the cells were mock treated, infected with SeV, or treated with doxorubicin, and 20 h later, luciferase activity was measured (Fig. 5A). Each cell line responded comparably to SeV infection, regardless of the form of cGAS expressed. In the cells lacking cGAS but possessing STING, a modest upregulation of the IFN-β promoter was seen in response to doxorubicin. IFN-β activation by doxorubicin was substantially enhanced by expression of cGAS-wt when STING was present. In neither the cGAS-NTM nor the cGAS-DBM cells did doxorubicin result in the enhanced IFN-β promoter activation (Fig. 5A). This suggests that the enhanced IFN-β response to doxorubicin requires cGAS with an intact DNA-sensing capacity and STING and that STING is responding to cGAS-generated CDN. This provides evidence that doxorubicin can activate the cGAS-STING DNA-sensing pathway to induce IFN-β expression. In these experiments, VP35 expression inhibited the SeV-mediated but not the doxorubicin-mediated activation of the IFN-β promoter, indicating that VP35 is unable to block doxorubicin-induced signaling through cGAS and STING.

To determine whether ATM signaling contributes to the cGAS-STING response, a 293T-based stable reporter cell line expressing cGAS-wt and STING was generated (cGAS-wt+STING-FF cells). The control or the STING+cGAS-wt reporter cell line was then transduced with either an empty-vector lentivirus or a VP35-expressing lentivirus. Three days after transduction, the cells were mock treated or treated for 2 h with ATM kinase inhibitor (Fig. 5B). Alternatively, the cells were transfected with expression plasmids that produce either a scrambled shRNA or an ATM-specific shRNA (Fig. 5C). Two days later, these cells were then mock treated, infected with SeV, treated with doxorubicin, or transfected with cyclic di-GMP (c-di-GMP), a CDN that can activate signaling through STING. Twenty hours later, luciferase activity was determined. The c-di-GMP treatment stimulated the IFN-β promoter in cGAS-wt+STING-FF cells relative to control cells lacking STING and cGAS (Fig. 5B and C), demonstrating that STING signaling is intact. Expression of STING and cGAS once again significantly enhanced doxorubicin-mediated IFN-β promoter activation (Fig. 5B and C). The presence of ATM kinase inhibitor or ATM shRNA dampened but did not abolish this response (Fig. 5B and C). This suggests that the ATM pathway contributes to the response in the presence of STING and cGAS. As seen previously, VP35 inhibited the SeV-induced response. However, VP35 did not inhibit the doxorubicin-induced response, the cyclic-di-GMP-induced response, or the residual IFN response detected in the presence of ATM inhibitor or ATM shRNA (Fig. 5B and C). This reinforces the view that ATM- and cGAS/STING-dependent IFN responses are insensitive to inhibition by VP35.

In humans, loss-of-function mutations in ATM result in ataxia telangiectasia (AT) (67–69). Comparison of fibroblasts from healthy subjects with ATM patient-derived fibroblasts demonstrated a constitutive elevation of IFN-β and ISG54 mRNAs in the absence of ATM, even when VP35 was introduced via a lentiviral vector (Fig. S2B). This is consistent with previous reports that sustained ATM deficiency upregulates an IFN response in AT cells via the STING pathway due to accumulation of damaged DNA in the cytoplasm (22). Interestingly, despite the fact that our transient knockdown of ATM decreased IFN responses to doxorubicin in the 293T cell system, treatment of AT fibroblasts with doxorubicin still yielded a strong IFN-β response compared to healthy control fibroblasts (Fig. S2C and D). The c-di-GMP treatment was used as a control for STING-mediated induction of IFN in these cells, which indeed resulted in modest activation of the IFN response. Notably, VP35 had no inhibitory effect on c-di-GMP- or doxorubicin-mediated response in these cells. This demonstrates that doxorubicin can induce an IFN response in the absence of ATM and that doxorubicin therefore can induce IFN-β by at least two distinct pathways, neither of which is impaired by VP35.

To confirm IFN induction in cells that express endogenous cGAS and STING and that have a responsive ATM signaling machinery, primary human monocyte-derived dendritic cells (MDDCs) were examined (Fig. S2E and F) (70). MDDCs were transduced with control or VP35 lentiviruses and 72 h later infected with SeV or treated with either doxorubicin or c-di-GMP. RNA was then isolated to determine IFN-β (Fig. S2E) and ISG54 (Fig. S2F) mRNA levels. Again, doxorubicin upregulated the IFN response in either the presence or the absence of VP35.

Because cGAS detects DNA (29) and because DNA damage can result in accumulation of single-stranded DNA (ssDNA) and double-stranded DNA (dsDNA) species in the cytoplasm (22), we asked whether doxorubicin treatment leads to colocalization of DNA and cGAS. We performed colocalization studies with ssDNA and cGAS. Indeed, ssDNA levels increased upon doxorubicin treatment, and this ssDNA colocalized with cGAS in both the nucleus and the cytoplasm (Fig. S3A and B). This suggests that doxorubicin-mediated DNA damage results in production of ssDNA that may directly activate cGAS.

10.1128/mBio.00368-17.4 FIG S3  Related to Fig. 5. Activation of IFN response by DNA generated by doxorubicin. (A) Colocalization of cGAS with ssDNA. HeLa cells were transfected with vector or cGAS. The next day, cells were mock treated or treated with doxorubicin (doxo, 1 μM) for 4, 8, or 12 h. Cells were fixed, processed for immunofluorescence, and analyzed by confocal microscopy. cGAS was detected using anti-FLAG antibody and ssDNA using anti-ssDNA with Alexa Fluor 488- and 647-labeled secondary antibodies, respectively (as shown in the green and red panels). Nuclei were stained with 4′,6-diamidino-2-phenylindole (DAPI) (blue). Bars, 10 µm. The insets show the zoomed-in images of cGAS and ssDNA colocalization regions. (B) The fluorescence intensity profiles for confocal images. The line scans of confocal images showing the overlap of peaks among cGAS, ssDNA, and DAPI as indicated with black arrows (red, green, and blue, respectively). The insets in panel A were used for quantification. (C) Effect of Trex1 expression on doxorubicin-mediated IFN activation. The control or STING-FF cells were transfected with vector or cGAS and also with Trex1 or Trex1 D18N mutant. The next day, cells were treated with doxorubicin (doxo, 1 μM) or SeV or transfected with interferon stimulatory DNA (ISD; 30 μg/ml) for 24 h. The following day, cells were lysed and luciferase activities were measured. Download FIG S3, PDF file, 0.4 MB. Copyright © 2017 Luthra et al. 2017 Luthra et al. This content is distributed under the terms of the Creative Commons Attribution 4.0 International license.

To further evaluate whether a DNA signal is responsible for the doxorubicin-stimulated IFN response, we overexpressed Trex1, a 3′ exonuclease that degrades the single- and double-stranded DNA in the cytoplasm and that can prevent activation of STING (57, 71, 72). Overexpression of Trex1 abrogated activation of IFN by doxorubicin and by exogenously delivered immunostimulatory DNA (ISD) mediated in the presence of cGAS-STING (Fig. S3C). As a control, we also overexpressed a Trex1 dominant mutant, D18N, that lacks the ability to degrade dsDNA and is associated with autoimmune disorders (71, 73, 74). Expression of the D18N mutant enhanced IFN response to doxorubicin above that seen in the absence of the mutant, suggesting that doxorubicin treatment results in cytoplasmic DNA that can trigger activation of the cGAS-STING pathway (Fig. S3C). Interestingly, in the control cells which lack cGAS and STING expression, a basal level of IFN activation was detectable following doxorubicin treatment but not following treatment with ISD, and this induction was not affected by Trex1 expression. This further supports the view that doxorubicin activates interferon through both STING-dependent and -independent pathways. It also suggests that the STING-independent pathway does not require the generation of cytoplasmic DNA.

### Doxorubicin inhibits Ebola virus infection *in vitro.*

**FIG 6:**
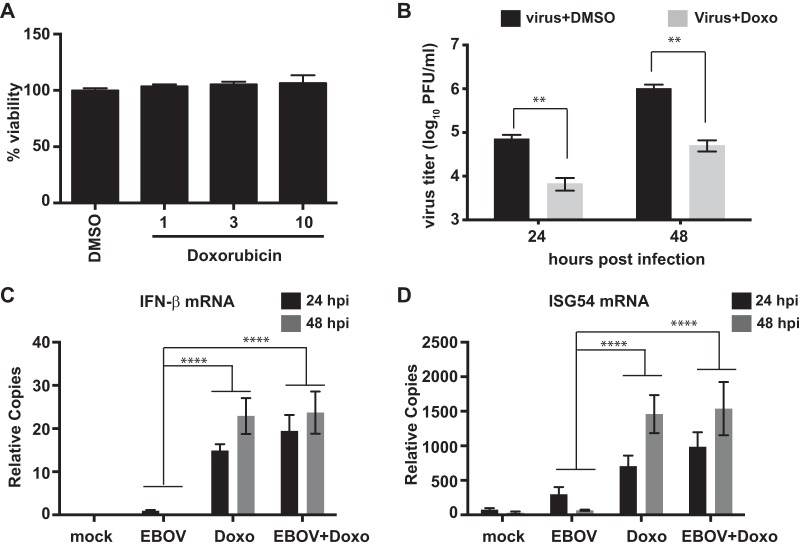
Effect of doxorubicin *in vitro*. (A) The toxicity of doxorubicin was evaluated in A549 cells using the CellTiter-Glo assay at 48 h after treatment with the drug. (B) Titers of an Ebola virus that expresses GFP (EBOV) after infection at a multiplicity of 2 of A549 cells in the presence of DMSO or doxorubicin (10 μM) (Doxo). The cells were pretreated with doxorubicin prior to infection for 1 h, and doxorubicin was added back to the medium after the infection. The error bars indicate the standard deviations from three independent replicates. **, *P* value < 0.01 (Student’s two-tailed *t* test). Data represent means ± standard deviations from two independent experiments (each performed in triplicate). (C and D) qRT-PCR for endogenous IFN-β (C) and ISG54 (D) mRNA levels normalized to β-actin mRNA at indicated postinfection time points. The error bars indicate the standard deviations from three independent replicates. ***, *P* value < 0.001; ****, *P* value < 0.0001 (Student’s two-tailed *t* test). hpi, hours postinfection.

Because doxorubicin can induce an IFN response in the presence of VP35, the antiviral activity of doxorubicin toward EBOV was assessed following drug pretreatment and infection of A549 cells (multiplicity of infection [MOI] of 2). Cell cytotoxicity was assessed in parallel on uninfected cells by measuring ATP content. A 10 μM concentration of doxorubicin caused little or no cell death (Fig. 6A) but did significantly reduce replication of an EBOV expressing GFP (EBOV-GFP) by 20-fold, to 5 × 10^4^ PFU/ml from 1 × 10^6^ PFU/ml at 48 h (Fig. 6B). Consistent with its activation of IFN responses in the presence of VP35, EBOV-GFP infection had no impact on doxorubicin-induced IFN-β or ISG54 mRNAs in infected cells (Fig. 6C and D). Overall, these results suggest that doxorubicin is capable of stimulating an IFN response that has an anti-EBOV effect *in vitro*.

### Doxorubicin bypasses the IFN antagonists of multiple negative-sense RNA viruses.

**FIG 7:**
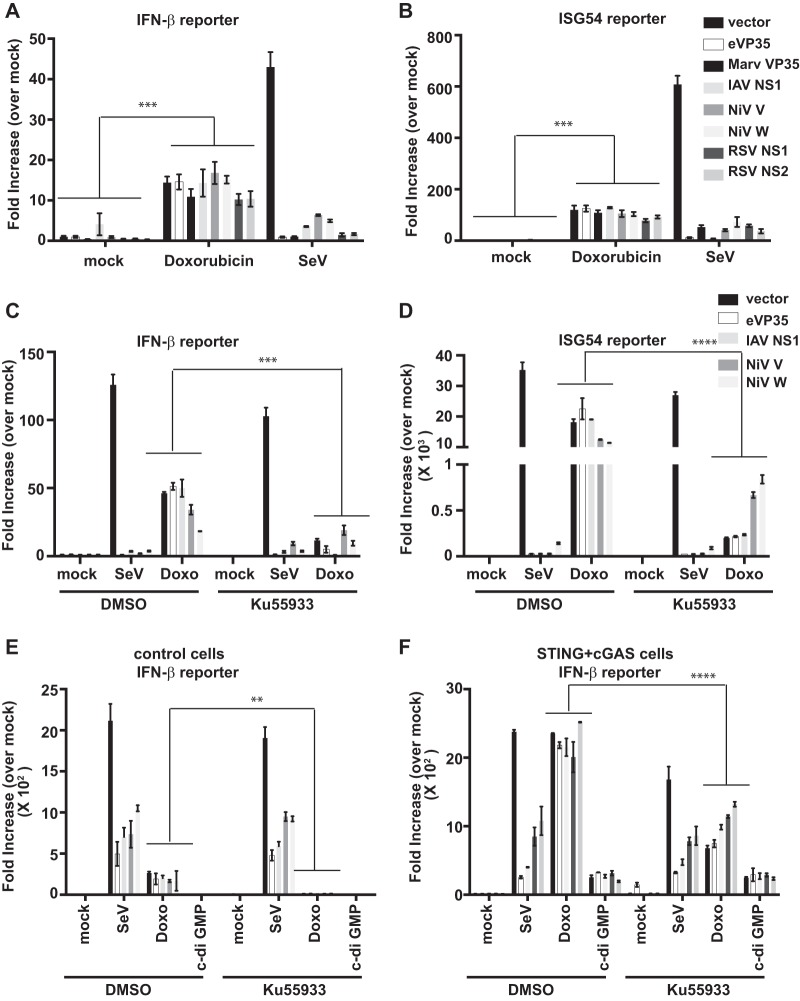
Doxorubicin bypasses multiple RNA virus IFN antagonists. IFN-β promoter (A) or ISG54 promoter (B) firefly luciferase reporter gene assays were performed. The empty vector (vector) or expression plasmids for the indicated viral IFN antagonists were transfected. The next day, cells were mock treated, treated with doxorubicin, or infected with SeV. Eighteen hours later, luciferase activity was determined. Fold induction was determined by setting the mock-treated (medium + DMSO) empty-vector controls to 1. Error bars indicate standard deviations from three independent replicates. Experiments similar to those described for panels A and B were performed to detect IFN-β promoter (C) or ISG54 promoter (D) reporter gene activity but with ATM kinase inhibitor pretreatment. An experiment similar to that described for panel C was performed using either the control-FF cells (E) or the cGAS-wt-STING-FF stable IFN-β reporter cells described in the legend to Fig. 5B (F). Data represent means ± standard deviations and are representative of three independent experiments. Error bars indicate standard deviations from three independent replicates. **, *P* value < 0.01; ***, *P* value < 0.001; ****, *P* value < 0.0001 (one-way analysis of variance followed by Tukey’s test). See also Fig. S4 in the supplemental material.

To determine whether doxorubicin can generally induce an IFN response by mechanisms that bypass the inhibitory effects of negative-sense RNA virus-encoded IFN antagonists, reporter assays were performed in the presence of EBOV VP35, Marburg virus VP35, influenza A virus NS1, Nipah virus (NiV) V and W, or respiratory syncytial virus (RSV) NS1 or NS2 proteins. Each protein was detectably expressed following transfection (Fig. S4). Doxorubicin stimulated IFN-β and ISG54 promoter activity in the presence of each of these IFN antagonists, whereas activation by RIG-I-activating SeV was inhibited by each of them (Fig. 7A and B). The presence of cGAS and STING dramatically enhanced the IFN-β promoter activation by doxorubicin in the presence of all antagonists relative to the control cells lacking cGAS and STING (Fig. 7E and F). Also, consistent with an inability of these viral proteins to block cGAS-STING signaling, c-di-GMP induced an IFN-β response in the cGAS-STING cells that was not inhibited by any of the IFN antagonists (Fig. 7F). Finally, as was previously seen with EBOV VP35, the presence of an ATM inhibitor reduced but did not eliminate the doxorubicin-induced IFN response in cGAS-STING cells, and this residual activity was not suppressed by the transfected viral proteins (Fig. 7C to F). These data suggest that activators of the ATM and cGAS-STING pathways might be exploited as a general strategy to induce an antiviral state in cells infected by negative-sense RNA viruses.

10.1128/mBio.00368-17.5 FIG S4  Related to Fig. 7. Expression of interferon antagonists. (A and B) Expression of Ebola virus VP35 (eVP35), Marburg virus VP35 (mVP35), influenza A virus NS1 protein, Nipah virus V and W proteins (NiV V and NiV W), and respiratory syncytial virus NS1 and NS2 proteins (RSV NS1 and RSV NS2) for the luciferase experiments described for Fig. 7A and B. Expression is shown for the mock-treatment samples. The proteins were detected using anti-FLAG antibody, and anti-β-tubulin was used as the loading control. Download FIG S4, EPS file, 2.8 MB. Copyright © 2017 Luthra et al. 2017 Luthra et al. This content is distributed under the terms of the Creative Commons Attribution 4.0 International license.

## DISCUSSION

DNA damage responses can trigger innate immune responses (22, 38, 52). Doxorubicin is a widely used anticancer drug that induces DNA damage (42). Its ability to intercalate with DNA, inhibit type II topoisomerase, generate free oxygen radicals, and trigger the DNA damage response contributes to its cytotoxic and cytostatic effects (33). In addition, doxorubicin modulates immune and cytokine responses that may influence its anticancer effects. For example, activation of IFN-γ-induced Jak-STAT signaling and ISG induction contributes to its antitumor effects (23). However, the molecular basis by which doxorubicin triggers immune modulation has not been clearly elucidated.

Because VP35 IFN inhibition is critical for EBOV virulence, we established a novel HTS assay that would allow the identification of compounds that activate the IFN-β promoter in the presence or absence of VP35. A screen performed with the assay identified doxorubicin, daunorubicin, and other anthracyclines as reproducible inducers of IFN in the presence of VP35 and SeV infection. Because the compounds were subsequently found to induce an IFN response in the absence of SeV infection and in the absence or presence of VP35, it was concluded that they do not directly inhibit VP35 and that the IFN induction pathway that is activated bypasses the inhibitory effects of VP35. VP35 has been well characterized as an inhibitor of the RIG-I signaling pathway that responds to 5′-triphosphate RNAs, short dsRNAs, and other RNA species to induce IFN expression. Data also suggest that VP35 can inhibit signaling via a second RIG-I-like receptor, MDA5. Mechanisms of RLR pathway inhibition by VP35 include sequestration of RIG-I-activating dsRNAs by the C-terminal dsRNA binding domain of VP35, known as the interferon inhibitory domain (IID), and interaction of the IID with host protein PACT, a facilitator of RIG-I activation (11, 75–77). Additional mechanisms include interaction with the kinases TBK1 and IκB kinase ε (IKKε), resulting in inhibition of IRF-3 phosphorylation, and facilitation of IRF-7 SUMOylation to repress IFN gene transcription (78, 79). Induction of IFN responses by anthracyclines in the presence or absence of VP35 suggests that a non-RLR signaling pathway was activated.

Although doxorubicin and other compounds that cause DNA damage had previously been implicated as IFN inducers, proposed mechanisms differ between studies (21, 38, 67). One study implicated signaling through ATM, a serine/threonine kinase that phosphorylates numerous substrates and mediates signaling downstream of the DNA break to facilitate DNA repair. The results of our screen are consistent with the known capacity of doxorubicin, daunorubicin, and other such drugs to trigger a DNA damage response and to activate ATM, as they triggered the phosphorylation of ATM and its downstream target p53 on serine 15 (21). Further, activation of the IFN response by doxorubicin and daunorubicin was impaired by either of two inhibitors of the DNA damage response signaling pathway, ATM inhibitor Ku55933, which directly inhibits ATM, or mirin, which inhibits the Mre11-Rad50-Nbs1 (MRN)-ATM pathway. Doxorubicin induces dsDNA breaks by targeting topoisomerase 2 (Top2), leading to stabilized cleavable dsDNA breaks to which the enzyme is covalently bound. Downregulation of Top2A has previously been demonstrated to reduce doxorubicin-mediated cytotoxicity (34). Consistent with inhibition of Top2A as a trigger for the ATM-dependent IFN response, knockdown of Top2A in our cell lines that lack detectable cGAS and STING decreased the IFN response triggered by doxorubicin.

Because the cGAS-STING DNA-sensing pathway has been implicated in responding to DNA damage (22), we also investigated whether cGAS and STING can be activated by doxorubicin. When cGAS and STING were present, doxorubicin also triggered an IFN response by this pathway. cGAS recognizes cytosolic DNA derived from either pathogens or damaged host cells and induces an IFN response through the adaptor protein STING. cGAS binds DNA and catalyzes the synthesis of cGAMP. cGAMP, an endogenous second messenger, binds and activates signaling through STING (27, 28). According to published studies and our Western blotting data, 293T cells lack detectable cGAS or STING expression. We therefore stably introduced STING or both cGAS and STING into the reporter cell lines. The presence of cGAS and STING resulted in enhanced IFN responses to doxorubicin. The enhancement required the presence of STING and a form of cGAS able to recognize DNA and generate CDN, indicating that doxorubicin activates the DNA-sensing pathway to trigger IFN responses. In the cGAS- and STING-expressing cells, the ATM pathway still contributes to the response to doxorubicin as evidenced by the reduced IFN response in the presence of ATM inhibitor. Whether ATM and cGAS-STING act as separate pathways in which each induces the IFN-β response or whether they act within the same pathway remains to be determined.

**FIG 8:**
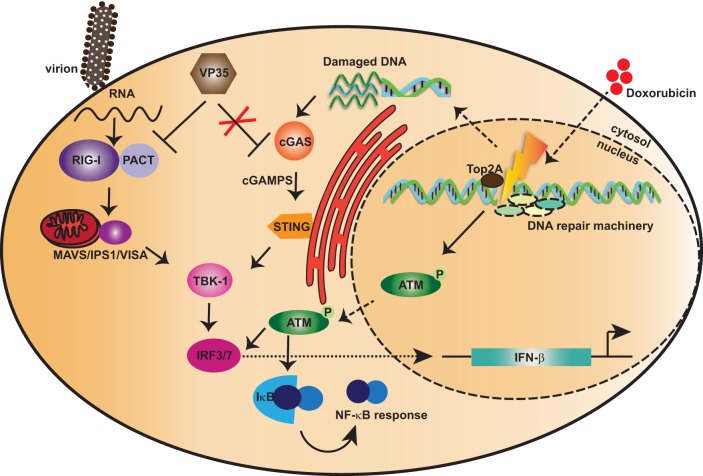
Proposed model for activation of IFN by doxorubicin bypassing IFN antagonism by Ebola virus VP35 protein. Ebola virus VP35 antagonizes IFN responses triggered by RIG-I-like receptors (RLR), which include RIG-I and melanoma differentiation-associated protein 5 (MDA5). RLR detect cytoplasmic double-stranded RNAs (dsRNAs) or RNAs with 5′ triphosphate (5′ pppdsRNA), products of RNA virus replication. The activation of RLR is further facilitated by protein kinase R activator (PACT). Upon activation, RLR signal through the mitochondrial antiviral signaling protein (MAVS) to activate kinases IκB kinase ε (IKKε) and TBK1. These kinases phosphorylate IFN regulatory factor 3 (IRF-3) or IRF-7, which then accumulates in the nucleus and promotes expression of type I IFNs. Doxorubicin treatment results in IFN induction by two independent pathways: the DNA damage repair response pathway involving ATM and DNA sensor machinery cGAS-STING. The DNA damage leads to activation of ATM that triggers activation of an IRF-3 and/or NF-κB response, thus leading to IFN activation. Furthermore, damaged DNA can also be detected by a cytoplasmic DNA sensor, cGAS, which through the STING–TBK1–IRF-3 axis leads to activation of IFN responses. Interestingly, these DNA-mediated IFN activation pathways are not subverted by the presence of Ebola virus VP35 protein. Thus, these data suggest novel avenues for developing antiviral therapeutics.

Cytoplasmic DNA triggers activation of the cGAS-STING pathway, so we evaluated if doxorubicin can induce production of cytoplasmic DNA. In our experiments, we detected increased abundance of ssDNA upon doxorubicin treatment that also colocalized with cGAS, implicating cGAS in IFN induction by doxorubicin. To further support the idea that accumulation of DNA is responsible for IFN induction by doxorubicin in the presence of cGAS-STING, we performed studies with Trex1. Trex1 is a 3′-5′ exonuclease responsible for degrading cytoplasmic DNA. Mutations in Trex1 cause the IFN-associated autoimmune disease Aicardi-Goutières syndrome (AGS) (80). Because IFN induction was abolished in the cGAS-STING cells when Trex1 was overexpressed, doxorubicin likely results in the accumulation of cytoplasmic ssDNA that activates cGAS-STING signaling to induce IFN production. Furthermore, we also noticed that in the absence of cGAS-STING, Trex1 overexpression has little or no effect on IFN activation, suggesting that doxorubicin-mediated IFN activation by the ATM pathway is not entirely dependent on cytoplasmic DNA production. dsDNA could also contribute to the observed IFN induction by doxorubicin via cGAS-STING, as we did not include or exclude its presence. Overall, our results indicate roles for both cGAS-STING and ATM in IFN induction by doxorubicin, a response that is not blocked by VP35 (Fig. 8). These data suggest that the ATM and cGAS-STING DNA-sensing pathways have potential as therapeutic approaches for EBOV because they bypass the inhibitory effects of VP35.

It has been demonstrated that activation of RLR pathways prior to EBOV infection can substantially suppress EBOV replication in cell culture (81). Further, recombinant EBOVs with mutant VP35s defective for dsRNA binding and RLR inhibition are highly attenuated in cell types that can mount an IFN response and in *in vivo* models of infection (12, 17, 82). Therefore, activation of IFN-inducing pathways that bypass VP35 inhibitory functions would also be expected to elicit an IFN response that can impair virus replication. Consistent with this, doxorubicin decreases EBOV growth in A549 cells at noncytotoxic concentrations. Induction of antiviral ISGs likely explains this suppression of EBOV growth. As shown in Fig. S2A in the supplemental material, cGAS and STING were not detected by Western blotting in A549 cells. Therefore, it is unclear to what extent cGAS-STING may contribute to the antiviral effects of doxorubicin in these cells. It is possible that cells with more robust cGAS-STING levels would demonstrate a more profound inhibition. *In vivo*, macrophages and dendritic cells are the primary target for EBOV infection (83, 84). These cell types also express cGAS and STING (85, 86), and our dendritic cell data (Fig. S2E and F) demonstrate the responsiveness of these cells to doxorubicin. Therefore, it seems likely that the *in vivo* cellular targets of EBOV will be responsive to this therapeutic approach.

Previous studies have demonstrated several mechanisms by which VP35 can impair activation of RLR signaling by RNA and RNA virus-triggered IFN production (8–11, 14). Inhibition correlates with VP35 dsRNA binding activity and VP35 interaction with host protein PACT, which facilitates activation of RIG-I by dsRNA or by virus infection (11, 75, 77). Studies in primary human dendritic cells (DCs) support these activities of VP35 in biologically relevant cell types and suggest inhibition of both RIG-I and the related RIG-I-like pattern recognition sensor MDA5 (87). In DCs, VP35 not only blocks IFN production but also decreases cytokine and chemokine production, inhibits upregulation of costimulatory markers, and impairs activation of T cells in response to virus infection (15). Therefore, activation of IFN responses via non-RLR pattern recognition receptors in EBOV-infected DCs might also promote activation of adaptive immune responses to EBOV. However, VP35 is also reported to impair activation of IRF-3 and IRF-7 by the kinases TBK1 and IKKε and to affect transcriptional activity of IRF-7 via effects on SUMOylation machinery (79). Therefore, why does it not still prevent induction of IFN responses by the ATM and cGAS-STING pathways? We hypothesize that inhibition at these downstream steps is relatively weak and can be overcome by relatively strong IFN-inducing signals. Consistent with this, in DCs, the relatively weak IFN response of cells to lipopolysaccharide (LPS) was impaired by wild-type or mutant VP35 (15). However, only wild-type VP35 could effectively impair the robust IFN response induced by SeV infection. This would also explain why mutations that abrogate VP35 dsRNA binding activity and inhibition of RLR activation but which do not impair VP35 inhibition of signaling by TBK1 and IKKε are sufficient to lead to severe attenuation of EBOV in cells and *in vivo*.

Given our data in the VP35-expressing cells, we hypothesized that the ATM and cGAS-STING pathways may provide a means to generally trigger antiviral responses in cells infected with negative-sense RNA viruses. Such viruses are well documented to encode inhibitors of RLR signaling and many also encode inhibitors of IFN-α/β-induced Jak-STAT signaling. However, such viruses may not have evolved mechanisms to counter DNA-triggered innate immune signaling pathways. The activation of such pathways may have multiple beneficial effects, such as inducing IFN and also triggering an intrinsic, IFN-independent antiviral response, for example, by activation of IRF-3, which can trigger antiviral gene expression independently of IFN production (38, 88, 89). Consistent with our hypothesis, doxorubicin induced an IFN response in the presence of IFN-antagonist proteins from Marburg virus, influenza A virus, Nipah virus, and respiratory syncytial virus. Thus, the data in this study suggest the ATM and cGAS-STING pathways as novel avenues to develop new broad-spectrum antivirals.

## MATERIALS AND METHODS

### Cell lines.

293T and A549 cells were obtained from the American Type Culture Collection (ATCC) (http://www.atcc.org) and maintained in Dulbecco’s modified Eagle’s medium (DMEM) (Gibco) supplemented with 10% fetal bovine serum (FBS; HyClone) and penicillin-streptomycin (Gibco). Human healthy fibroblasts (GM05294; Coriell Institute for Medical Research) and AT patient fibroblasts (GM02052; Coriell Institute for Medical Research) were maintained in DMEM containing 10% (vol/vol) fetal calf serum (FCS; HyClone). Human monocyte-derived dendritic cells (MDDCs) were generated from CD14^+^ cells purified from concentrated leukocytes of healthy human donors (New York Blood Center), as described previously (15). For additional information on cell lines, please see the supplemental material.

### HTS.

High-throughput screening (HTS) was performed at the Mount Sinai Integrated Screening Core. To evaluate the robustness of the assay, we calculated the Z factor (27) and signal-to-background (S/B) ratio.

### Luciferase reporter gene assays.

HEK293T cells were transfected by using Lipofectamine 2000 (Invitrogen) with the indicated expression plasmids along with the reporter plasmids. At 20 h posttransfection, the cell lysates were assayed with the dual luciferase reporter assay (Promega), and firefly luciferase activity was normalized to *Renilla* luciferase activity.

### Monitoring cellular interferon responses.

To monitor activation of IFN responses, cells were either treated with doxorubicin (1 µM or 3 µM) or TNF-α (20 ng) or transfected with c-di-GMP (20 µg/ml) using LyoVec (InvivoGen) for the indicated times. SeV (Cantell strain) stocks were prepared by growth in 10-day-old embryonated chicken eggs for 2 days at 37°C, and infections were performed as described in Results.

### RNA extraction and qRT-PCR for cellular mRNAs.

The total RNA was isolated from the cells using Trizol according to the manufacturer’s instructions. cDNAs were synthesized using the SuperScript III First-Strand synthesis system (Invitrogen). The resulting cDNAs were used as the templates for subsequent quantitative PCRs using gene-specific primers (IFN-β, ISG54, IFN-α, or β-actin).

### EBOV-GFP infection assays.

All experiments using infectious EBOV were performed in biosafety level 4 (BSL-4) facilities of the Galveston National Laboratory. The viral titers were determined by plaque assay. The cell viability in infection experiments was determined using the Viral ToxGlo assay (Promega), and ATP content was determined by reading luminescence using a BioTek Synergy HT plate reader.

10.1128/mBio.00368-17.1 TEXT S1  Supplemental experimental procedures and supplemental references. Download TEXT S1, DOCX file, 0.1 MB. Copyright © 2017 Luthra et al. 2017 Luthra et al. This content is distributed under the terms of the Creative Commons Attribution 4.0 International license.
