# Supplementary figures and images for: Topoisomerase II Inhibitors Induce DNA Damage-Dependent Interferon Responses Circumventing Ebola Virus Immune Evasion
Source: mBio. 2017 Apr 4;8(2):e00368-17. doi: 10.1128/mBio.00368-17 (PMC5380843; doi:10.1128/mBio.00368-17)

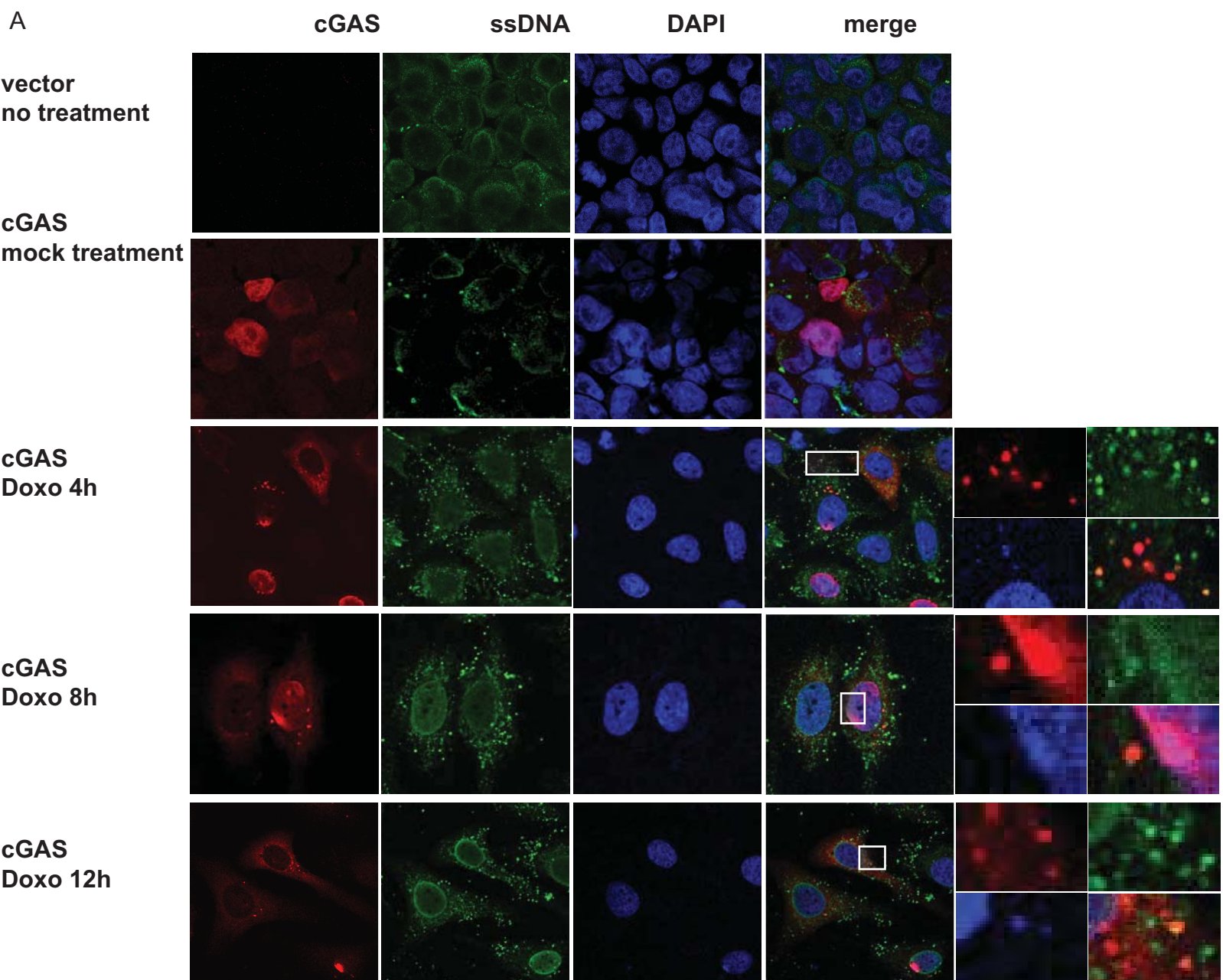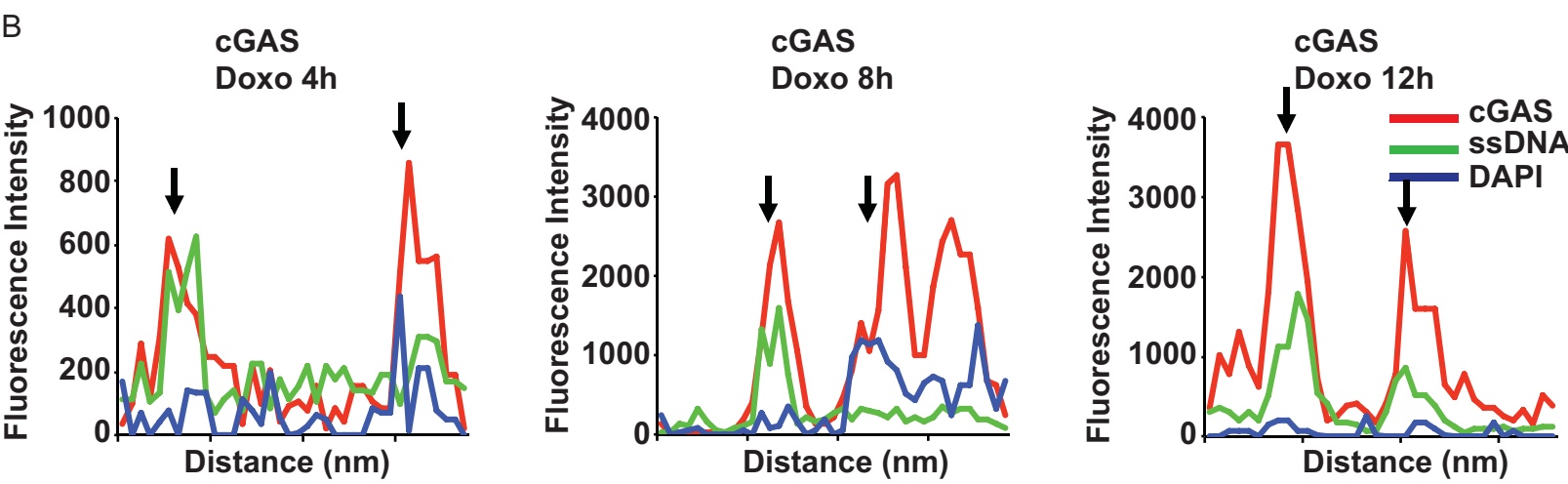

C

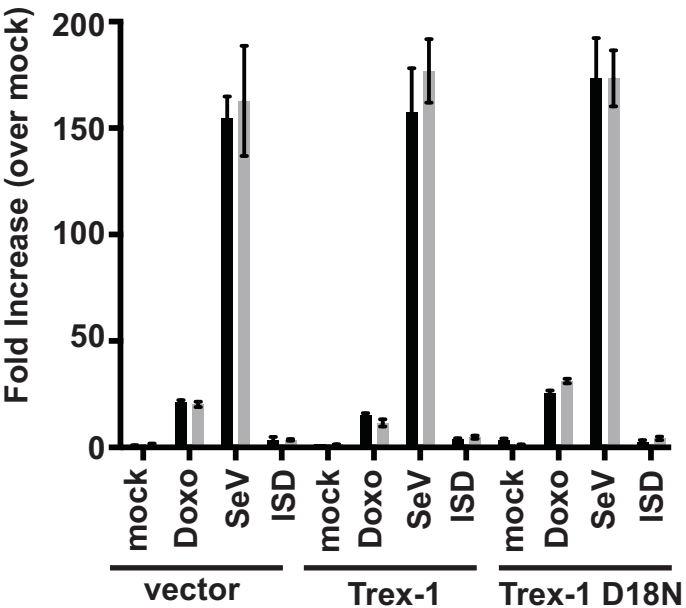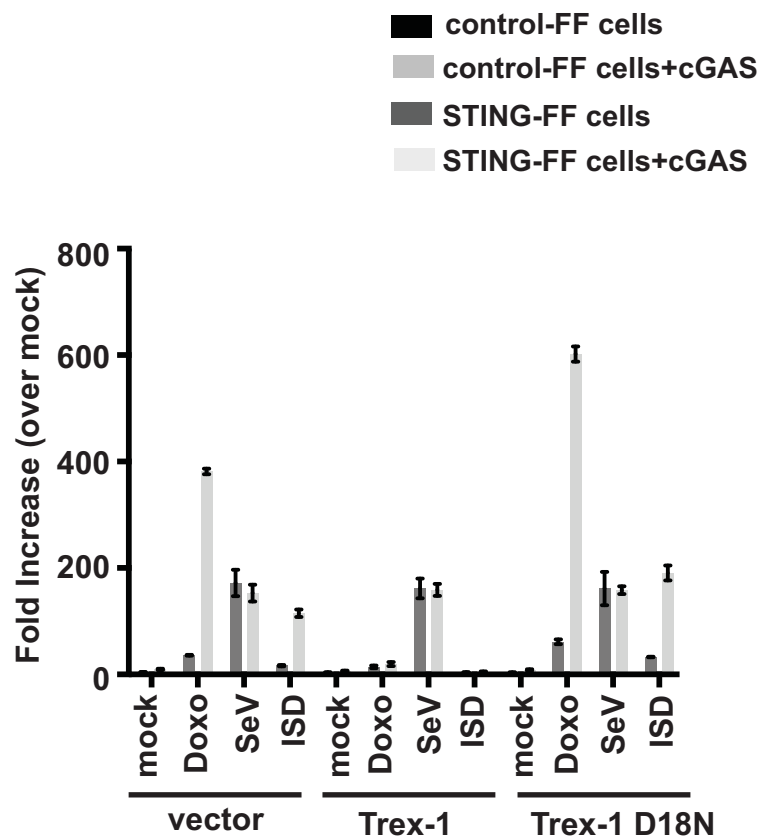

Supplement: FIG S3 [file mbo002173264sf3.pdf]
