# Supplementary material for: Topoisomerase II Inhibitors Induce DNA Damage-Dependent Interferon Responses Circumventing Ebola Virus Immune Evasion
Source: mBio. 2017 Apr 4;8(2):e00368-17. doi: 10.1128/mBio.00368-17 (PMC5380843; doi:10.1128/mBio.00368-17)
Supplement: TEXT S1 [file mbo002173264s1.docx]

**Supplemental Materials**

**Experimental Procedures and Supplemental References**

**Experimental Procedures**

**Generation of lentiviral expression vectors and stable cell lines**

Replication-deficient lentiviruses were generated as previously described^1,2^. The following expression plasmids were used to generate lentivirus vector-derived virus like particle (VLPs): pHCMV-G encoding the vesicular stomatitis virus (VSV) glycoprotein; packaging plasmid pNL4.3-gag-pol; and VP35 or STING-encoding lentiviral vectors derived from pHR-SIN-CSIGW ^1,3^. The Flag tagged VP35 or STING lentivirus vector contains the EBOV VP35 or STING open reading frame (ORF) upstream of an internal ribosomal entry site, which is followed by green fluorescent protein (GFP). The control lentivirus is the same except that it only encodes GFP.

Cells were sorted based on GFP expression. Individual clones were isolated, expanded and tested for reporter gene expression upon SeV infection. The selected clone, based on effective suppression of IFNβ promoter activation, was maintained in DMEM supplemented with 10% fetal bovine serum, 1% penicillin-streptomycin and 2 µg/mL of Geneticin. The single clonal reporter cell lines were obtained; control-FF, VP35-FF and STING-FF cells.

Stable VP35-expressing or control cells were generated by transducing a parental 293T cell line that contains a firefly luciferase gene under the control of the IFN-β promoter (293T-FF) ^4^ with replication-defective lentiviruses that express VP35 and GFP (VP35-FF cells) or only GFP (control-FF cells).

Stable STING-expressing reporter cells (STING-FF cells) were generated in a similar manner. The 293T-FF cells were transduced with lentiviruses expressing human STING with an arginine at position 232, which is responsive to canonical CDNs as well as cGAS-generated CDNs.

The cGAS and STING expressing reporter cells (cGAS-wt+STING-FF cells) were generated by transfecting plasmids expressing cGAS-wt in STING-FF cells and selecting with puromycin (2µg/ml). Individual clones were selected and amplified in the presence of puromycin.

**Preparation of human monocyte derived dendritic cells (MDDCs)**

Peripheral blood mononuclear cells (PBMCs) were isolated by Ficoll (GE Healthcare) density gradient centrifugation of buffy coats from anonymous human donor blood bank blood, and CD14^+^ cells were purified by using CD14 microbeads (Miltenyi Biotech). CD14^+^ cells, at a concentration of 1x10^6^ cells/ml, were incubated at 37°C for 5 days in DC medium (RPMI containing 4% human AB serum [Fisher Scientific], 2mM L-glutamine, 1mM sodium pyruvate, 100 U/ml penicillin–100μg/ml streptomycin, and 55μM β-mercaptoethanol) supplemented with 500 U/ml human granulocyte-macrophage colony-stimulating factor (hGM-CSF; PeproTech) and 500 U/ml human interleukin-4 (hIL-4; PeproTech). Typical yields are approximately 10^7^ monocyte derived dendritic cells (MDDCs) per 20 ml of culture.

**Plasmids, Antibodies and Other Reagents**

A mammalian expression plasmid for cGASwt in pCMV6 was purchased from Origene. The cGAS NTPase mutant (NTM), and cGAS DNA binding mutant (DBM) were cloned with C-terminal FLAG tags into the mammalian expression plasmid pCMV6. The STING plasmid has been described ^5,6^ and for this study the expression plasmid for human STING with an H232R change was cloned in the pTR600 vector. ATM shRNA, scrambled shRNA plasmids (in pSUPER-CFP vector background), Trex-1 and Trex-1 D18N plasmids were kindly provided by Dr. Lubbertus Mulder (Icahn School of Medicine at Mount Sinai). ATM plasmids are previously described ^7^. The pCAGGS-VP35 plasmid has been previously described ^8^. Expression plasmids for V and W proteins of Nipah virus and NS1 of influenza A virus were described previously ^9^. Expression plasmids for the human respiratory syncytial virus proteins were cloned into pCAGGS ^10^.

The pRL-TK Renilla luciferase expression plasmid was purchased from Promega (Madison, WI). pCAGGS-firefly luciferase, IFN-β reporter, NF-κB reporter plasmid, and ISG54 reporter plasmids have been previously described ^8^.

Monoclonal antibodies to EBOV VP35 have been described previously ^8,11^. Monoclonal anti-hemagglutinin (anti-HA) and anti-FLAG epitope antibodies were purchased from Sigma (St. Louis, MO). Rabbit polyclonal anti-ATM antibody was purchased from Cell Signaling (Boston, MA). Monoclonal mouse anti-phospho ATM (ser1981) antibody was purchased from Santa Cruz Biotechnology (Dallas, TX). Rabbit monoclonal anti-phospho-IRF-3 (S396) (4D4G) antibody was purchased from Cell Signaling and rabbit polyclonal anti-IRF3 antibody was purchased from Santa Cruz Biotechnology. Monoclonal mouse anti-FLAG antibody conjugated to magnetic beads was purchased from Sigma. Monoclonal antibody anti-P53 and anti-phospho P53 (ser15) was purchased from Cell Signaling Technology.

Recombinant human IFN-β was purchased from Calbiochem (San Diego, CA). Cyclic-di-GMP was purchased from InvivoGen (San Diego, California). ATM kinase inhibitor KU55933 and MRN-ATM pathway inhibitor mirin were purchased from Santa Cruz Biotechnology.

**High throughput screening (HTS)**

HTS was performed at the Mount Sinai Integrated Screening Core. HTS was performed in duplicate using Costar solid white 384-well plates. The plates were seeded with 20μl of VP35-FF cells (clone 3s) (25,000/well) in DMEM without phenol red supplemented with 10% FBS using a Multidrop Combi Reagent Dispenser (Combi) (Thermo Scientific). The cells were allowed to rest in a 37°C incubator with 5% CO_2_ condition for 2hrs. SeV diluted in 10% FBS DMEM (phenol red free media, 1:1000 dilution, negative control) or 10μl of SeV plus doxorubicin (3μM) as a positive control was added to the wells. Doxorubicin was initially chosen as a positive control because it is an FDA approved drug that had been previously reported to induce activation of the interferon pathway ^12-15^. Two columns in each plate were used as negative and positive controls. Compounds (30nl, final concentration 7.5-8μM) were added to plates by pintool (V&P Scientific) transfer. Twenty hours later, 15μl of Neolite luciferase assay reagent (Perkin Elmer) was added to each well by use of a Combi reagent dispenser and the luciferase signal was measured by using an EnVision plate reader (PerkinElmer). Z-Factor values for each plate were calculated using the equation Z-factor = 1 −[(3σc+ + 3σc−)/(|μc+ − μc−|)]; σ is the standard deviation and the μ is the mean. “c+” is the positive control of SeV and doxorubicin; “c-” represents the negative control consisting of SeV and DMSO. S/B = μc+/μc−. The primary hits are identified by calculating the Z-score for each compound and applying hit selection criteria where a Z-score greater than or equal to 5 is scored as a hit. Z-scores for each compound were calculated using the equation Z-score = (Xcompound–μplate)/σplate; the plate mean (μ) and plate standard deviation (σ) were determined for individual plates from all wells treated with library compounds. Confirmation assays with the compounds were completed in 96 well plates (25k cells/50μL/well) and cells were treated as described for the HTS assays. Cell viability was assessed with CellTiter-Glo assay (Promega).

**Chemicals for screening**

The chemical library used for the screen at the Mount Sinai Integrated Screening Core was purchased from Microsource Discovery (Gaylordsville, CT, USA) and contains 2080 bioactive compounds. Doxorubicin and daunorubicin were purchased from Sigma-Aldrich. All chemicals were adjusted to create a stock solution of 20mM in DMSO before use and were diluted to the desired concentration as indicated in the figures.

**Cell viability assay**

HEK293T cells (1x10^3^ cells/50μL/well) were plated in 96-well plates (white opaque Culture Plate, Costar). The cells were treated with compounds as described for the confirmation and high throughput assays. Two hours after plating, cells were mock infected with SeV (50μL) and the compounds were added to reach the indicated final concentrations (0−100μM, in a 2-fold dilution series). Twenty hours post-treatment CellTiter-Glo (20μL, Promega) was added, and ATP content, a measure of cell viability, was determined by reading luminescence using a GlomaxMulti+Microplate (Promega) reader. The assays were performed in triplicate.

**Luciferase reporter gene assays**

HEK293T cells were transfected by using Lipofectamine 2000 (Invitrogen) with the indicated expression plasmids and an NF-κB-promoter, IFNβ-promoter or ISRE firefly luciferase reporter plasmid (100ng). A constitutively expressed *Renilla* luciferase reporter plasmid, pRLTK (10 ng), was co-transfected as a control for transfection efficiency. At 20hr post-transfection, the cell lysates were assayed with the Dual Luciferase reporter assay (Promega) and firefly luciferase activity was normalized to *Renilla* luciferase activity.

ATM kinase Inhibitor (10 µM) or Mirin (10 µM) treatments were performed as described in the Results. The cells were either infected with SeV or treated with doxorubicin as indicated in the figure legends. The c-di-GMP was introduced into cells by using LyoVec (Invivogen). The luciferase assays performed on the stable firefly reporter cell lines used Neolite luciferase substrate (Perkin Elmer).

The lysates were also run on 10% NuPAGE® Bis-Trispolyacrylamide gels or 4-8% clear PAGE SDS gels (for ATM), transferred to a PVDF membrane and probed with antibodies as indicated on the figures.

**Small interfering RNAs and shRNAs**

The control or VP35 reporter cells were transfected with pSUPER-CFP-plasmids encoding RNAi targeting ATM (ATM-sh, ACTGTAAAGCTGCAATGAA) or scrambled RNAi (SCRB-sh, CATGCCTGATCCGCTAGTC) using lipofectamine 2000. Forty eight hours later, the cells were treated with drugs or infected with SeV. Eighteen hours post-infection or drug addition, luciferase activity was measured using Neolite reagent.

The VP35 siRNA (VP35 219 and VP35 349) duplexes were synthesized by Dharmacon (Chicago, IL, USA) and correspond to previously described sequences ^16^. The control siRNA or VP35 siRNA was transfected into the stable VP35-FF cells using oligofectamine (Promega). Seventy-two hours later, the cells were treated with drugs or infected with SeV. Eighteen hours later, luciferase activity was measured using Neolite reagent.

Topa IIa specific siRNA was obtained from Santa CruzBiotechnology (sc-36695) and Topo IIa (F-12) (sc-365916, Santa Cruz Biotechnology) antibody was used to monitor expression of Topo IIa.

**RNA extraction and qRT-PCR for cellular mRNAs**

The total RNA was isolated from the cells using Trizol following the manufacturer’s instructions. The isolated RNA was treated with 1 U of RNase-free DNase I (Ambion) for 30 min at 37°C. The reaction was terminated by using inactivation reagent and the cleared RNA was collected. The purified total RNA was reverse transcribed into cDNA using SuperScript III First-Strand synthesis system (Invitrogen). For cDNA synthesis oligo (dT) primers were used. The resulting cDNAs were used as templates for subsequent quantitative PCR reactions using gene specific primers (IFN-β, ISG54, OAS1or β-actin) and PerfeCTa® SYBR® Green FastMix® RT-PCR kits (Quanta Bioscience). Real-time PCR analysis was carried out using the BioRad 1000C Thermal Cycler. The threshold cycle (**C_T_**) values for the RNAs were normalized to the **C_T_** values of β-actin mRNA and the relative copy numbers were determined by using the formula, 2^-[^**^CT^**^(target gene)-^ **^CT^**^(beta-actin)]^. The quantitative reverse transcription-PCRs (RT-PCRs) were performed in triplicate.

**Immunoblotting**

For detection of total and phospho-IRF3, HEK293T cells were transfected using Lipofectamine 2000 (Invitrogen) with indicated plasmids, vector or eVP35 (500ng) and IRF3 (100ng). Eighteen hours post-transfection, cells were infected with SeV to activate the IFNβ pathway. Eight hours post- infection, cells were lysed with Nonidet P-40 lysis buffer [50mM Tris (pH 7.5), 280mM NaCl, 0.5% Nonidet P-40, 0.2mM EDTA, 2mM EGTA, 10% glycerol, protease inhibitor (cOmplete; Roche), and phosphatase inhibitor (PhosStop; Roche)]. Cells were incubated on ice for 20 min and then centrifuged at 14,000×g for 10 min at 4°C, and supernatants were collected. The cell lysates were run on 10% NuPAGE® Bis-Tris gels polyacrylamide (Thermo Fisher Scientific) and transferred to a polyvinylidenedifluoride membrane. The membrane was probed with anti-IRF3, anti–phospho-IRF3 (Ser396), anti-VP35, and anti-FLAG M2 and developed using Western Lightning ECL kit (Perkin-Elmer) and imaged using Bio-Rad chemi-doc MP system.

To detect total and phospho-ATM and p53, the cells were transfected as described above. The cell lysates were prepared using cold RIPA buffer [25mM Tris•HCl pH 7.6, 150mM NaCl, 1% NP-40, 1% sodium deoxycholate, 0.1% SDS] supplemented with protease and phosphatase inhibitors. After the 20 minutes incubation of lysates on ice, the lysates were sonicated for 30 seconds with a 50% pulse. The cells lysates were run on 4-8% clear PAGE SDS gels for ATM and p-ATM expression while for p53 and p-p53 on 10% NuPAGE® Bis-Trispolyacrylamide gels (Thermo Fisher Scientific).

**EBOV-GFP infection assays**

A549 cells (8x10^4^ cells cells/well) were plated in 6-well plates (Costar) overnight, and the following day, treated with doxorubicin (10μM) or DMSO (mock treatment). One-hour post-treatment, the cells were mock-infected or infected with EBOV-GFP ^17^ at an MOI of 2. The cells were then washed three times with Dulbecco’s Phosphate Buffered-Saline (DPBS, Corning Cellgro) and fresh media with or without the compounds was added to the cells. The compounds were left on the cells for the remainder of the experiment. The infection was performed in triplicate. At 24 and 48h post infection, the viral supernatant and the Trizol (Life Technologies) cell extracts were collected. To assess cell viability in infection experiments, 1x10^4^ A549 cells/well were plated in 96-well plates (white polystyrene, Costar) overnight and treated with compound as above. Cell viability was assessed 2 days after compound treatment using Viral ToxGlo (Promega) and ATP content was determined by reading luminescence using a BioTek Synergy HT plate reader.

**Immunofluorescence**

Hela cells were cultured and transfected with the empty vector or cGAS expressing plasmids with Lipofectamine 2000 (as per manufacturer´s protocol, Invitrogen) in 1.5 coverslips (MaTtek). Cells were fixed with 4% paraformaldehyde for 15 minutes and blocked with 1% bovine serum albumin (BSA) in PBS. Samples were incubated with the primary antibodies for 2h, washed three times with PBS and incubated for 1h with secondary antibodies. Nuclei were stained with 1 μg/mL DAPI (4,6-diamidino-192 2-phenylindole; Invitrogen).

**Microscope Image Acquisition**

Confocal laser scanning was performed using a Zeiss LSM 194 880 Meta (Carl Zeiss Microimaging, Thornwood, NY) fitted with a Plan Apochromatic 40x/1.4 oil objective. Images were collected at 12 bits and a resolution of 1024 by 1024 pixels. The confocal images were processed and analyzed with Fiji/Image J software.

**Statistical Methods**

For the screening assay, Z’ was used as statistical factor to define the variation in the assay and screening window. To select hits, the Z score was used, which defines how many standard deviations from the mean is a given value . The One-way ANOVA followed by tukey’s test was used to assess significance between multiple groups in reporter gene assays. Student’s two-tailed t test was used to identify significance between two unpaired groups. A p-value of <0.05 indicates a significant difference between experimental groups

**Supplemental References**

1. Aguirre, S.*, et al.* DENV inhibits type I IFN production in infected cells by cleaving human STING. *PLoS Pathog* **8**, e1002934 (2012).

2. Yen, B., Mulder, L.C., Martinez, O. & Basler, C.F. Molecular basis for ebolavirus VP35 suppression of human dendritic cell maturation. *J Virol* **88**, 12500-12510 (2014).

3. Demaison, C.*, et al.* High-level transduction and gene expression in hematopoietic repopulating cells using a human immunodeficiency [correction of imunodeficiency] virus type 1-based lentiviral vector containing an internal spleen focus forming virus promoter. *Hum Gene Ther* **13**, 803-813 (2002).

4. Martinez-Gil, L.*, et al.* Identification of small molecules with type I interferon inducing properties by high-throughput screening. *PLoS One* **7**, e49049 (2012).

5. Ishikawa, H. & Barber, G.N. STING is an endoplasmic reticulum adaptor that facilitates innate immune signalling. *Nature* **455**, 674-678 (2008).

6. Ishikawa, H. & Barber, G.N. The STING pathway and regulation of innate immune signaling in response to DNA pathogens. *Cell Mol Life Sci* **68**, 1157-1165 (2011).

7. McCabe, N.*, et al.* Deficiency in the repair of DNA damage by homologous recombination and sensitivity to poly(ADP-ribose) polymerase inhibition. *Cancer Res* **66**, 8109-8115 (2006).

8. Luthra, P.*, et al.* Mutual antagonism between the Ebola virus VP35 protein and the RIG-I activator PACT determines infection outcome. *Cell Host Microbe* **14**, 74-84 (2013).

9. Park, M.-S.*, et al.* Newcastle Disease Virus (NDV)-Based Assay Demonstrates Interferon-Antagonist Activity for the NDV V Protein and the Nipah Virus V, W, and C Proteins. *J. Virol.* **77**, 1501-1511 (2003).

10. Lo, M.S., Brazas, R.M. & Holtzman, M.J. Respiratory syncytial virus nonstructural proteins NS1 and NS2 mediate inhibition of Stat2 expression and alpha/beta interferon responsiveness. *J Virol* **79**, 9315-9319 (2005).

11. Leung, D.W.*, et al.* Structural basis for dsRNA recognition and interferon antagonism by Ebola VP35. *Nat Struct Mol Biol* **17**, 165-172 (2010).

12. Kurz, E.U., Douglas, P. & Lees-Miller, S.P. Doxorubicin activates ATM-dependent phosphorylation of multiple downstream targets in part through the generation of reactive oxygen species. *J Biol Chem* **279**, 53272-53281 (2004).

13. Kim, T.*, et al.* Activation of interferon regulatory factor 3 in response to DNA-damaging agents. *J Biol Chem* **274**, 30686-30689 (1999).

14. Hartlova, A.*, et al.* DNA damage primes the type I interferon system via the cytosolic DNA sensor STING to promote anti-microbial innate immunity. *Immunity* **42**, 332-343 (2015).

15. Brzostek-Racine, S., Gordon, C., Van Scoy, S. & Reich, N.C. The DNA damage response induces IFN. *J Immunol* **187**, 5336-5345 (2011).

16. Geisbert, T.W.*, et al.* Postexposure protection of non-human primates against a lethal Ebola virus challenge with RNA interference: a proof-of-concept study. *Lancet* **375**, 1896-1905 (2010).

17. Towner, J.S.*, et al.* Generation of eGFP expressing recombinant Zaire ebolavirus for analysis of early pathogenesis events and high-throughput antiviral drug screening. *Virology* **332**, 20-27 (2005).
